# Psychological Burden of Long COVID and Associated Factors Among Nurses Two Years Post-infection: A Cross-Sectional Study

**DOI:** 10.7759/cureus.99231

**Published:** 2025-12-14

**Authors:** Lin Zhang, Liang Li, Junyi Chen, Suhua Pang, Zhenjiang Zhang, Youde Yan

**Affiliations:** 1 Department of Infectious Diseases, The Affiliated Suqian First People’s Hospital of Nanjing Medical University, Suqian, CHN; 2 Department of Infectious Diseases, Xuzhou Medical University, Suqian Clinical College, Suqian, CHN

**Keywords:** anxiety, depression, long covid, nurses, occupational mental health

## Abstract

Background: The COVID-19 pandemic has generated sustained physical and psychological impacts on healthcare workers. Growing evidence suggests that some individuals develop persistent multisystem manifestations long after the acute phase, collectively referred to as long COVID. Nurses, who have borne heavy workloads and ongoing psychological stress since the pandemic, may be particularly vulnerable; however, limited research has examined their mental health status two years after infection.

Aims: This study aimed to evaluate anxiety and depression levels among nurses two years after COVID-19 infection, compare psychological characteristics across clinical departments, and identify factors associated with long COVID.

Methods: A cross-sectional online survey was conducted among nurses at a tertiary hospital, yielding 735 valid responses. Data were collected using a general information questionnaire, the Generalized Anxiety Disorder-7 (GAD-7), and the Patient Health Questionnaire-9 (PHQ-9). Participants were categorized into long COVID (n = 263) and non-long COVID (n = 472) groups. Between-group differences were examined using appropriate parametric or non-parametric tests, and variables with P < 0.10 were entered into multivariable logistic regression to identify independent predictors.

Results: Nurses with long COVID were significantly older and exhibited higher GAD-7 and PHQ-9 scores than those without long COVID (all P < 0.001). Multivariate analysis showed that higher PHQ-9 scores (odds ratio (OR) = 1.147, 95% confidence interval (CI): 1.108-1.188) and older age (OR = 1.040, 95% CI: 1.011-1.070) were independently associated risk factors for long COVID. Although the prevalence of long COVID did not differ significantly across departments (P = 0.378), anxiety and depression levels varied, with nurses in high-risk exposure departments reporting higher GAD-7 and PHQ-9 scores than those in outpatient and medical-technical departments.

Conclusion: Two years after infection, nurses with long COVID continue to experience substantial psychological burden, particularly among older individuals and those with more severe depressive symptoms. Although long COVID was evenly distributed across clinical departments, significant interdepartmental differences in anxiety and depression underscore the influence of work characteristics and environment. Targeted psychological support and ongoing mental health monitoring are warranted to promote recovery and enhance occupational resilience in the post-pandemic era.

## Introduction

The outbreak of coronavirus disease 2019 (COVID-19) at the end of 2019 has had a profound impact on global public health systems, socioeconomic stability, and population health [1,2]. Although most individuals recover after the acute phase, substantial evidence indicates that some patients develop long-term, multisystem dysfunction, manifested as persistent fatigue, respiratory symptoms, cognitive impairment, and other chronic complaints, lasting months to years after infection. This condition is collectively referred to as long COVID [3,4].

Nurses, as frontline healthcare professionals, have borne heavy workloads, infection risks, and sustained psychological stress throughout the pandemic and subsequent control phases [5]. Previous studies have demonstrated that healthcare workers, particularly nurses, face a significantly higher risk of anxiety, depression, insomnia, and other mental health problems compared with the general population. During the post-infection recovery phase, ongoing physical discomfort, occupational stress, and insufficient social support may further exacerbate emotional distress, contributing to chronic psychological burden [6,7].

Notably, while some studies have examined the psychological status of healthcare workers after COVID-19 infection, systematic investigations focusing on long COVID among nurses two years post-infection remain limited. This population not only continues to suffer from physical sequelae but also faces sustained occupational demands in high-intensity, fast-paced clinical settings, resulting in dual physical and psychological strain [8]. Differences in departmental characteristics, such as job nature, patient exposure risk, night-shift frequency, and workload intensity, may further contribute to heterogeneity in mental health outcomes [9]. Therefore, this study aimed to evaluate anxiety and depression levels among nurses two years after COVID-19 infection, compare psychological differences across departments, and identify factors associated with long COVID. The findings are expected to provide empirical evidence for long-term mental health surveillance, risk stratification, and targeted psychological interventions for nurses, supporting the establishment of a sustainable occupational mental health support system in the post-pandemic era.

## Materials and methods

Study design

This study adopted a cross-sectional design and conducted an online survey in June 2024 to assess anxiety and depression levels among nurses two years after confirmed COVID-19 infection. Participants were recruited using convenience sampling through internal communication platforms in participating hospitals. A structured online questionnaire incorporating validated psychological scales was used for data collection.

Participants

Inclusion Criteria

Participants were eligible if they were aged ≥18 years, had a confirmed history of COVID-19 infection at least two years prior, and provided voluntary informed consent.

Long COVID was determined using a predefined yes/no checklist of persistent physical symptoms lasting three months or longer after acute infection, based on commonly used World Health Organization (WHO) and National Institute for Health and Care Excellence (NICE) criteria. Participants without such symptoms were classified as the non-long COVID group, and all individuals in both groups had a confirmed COVID-19 history. To avoid overlap with psychological outcomes, mood-, sleep-, and cognition-related symptoms assessed by the Patient Health Questionnaire-9 (PHQ-9) and the Generalized Anxiety Disorder-7 (GAD-7) were excluded from the long COVID definition.

Exclusion Criteria

Participants were excluded if they had no confirmed history of COVID-19 infection; had severe pre-existing psychiatric disorders, cognitive impairment, or major comorbidities likely to affect psychological assessment; or declined to participate. Individuals with incomplete or invalid questionnaire responses were also excluded to ensure data quality.

Department Classification

To examine potential differences in psychological outcomes across clinical settings, nurses were classified into five departmental categories based on workplace characteristics and occupational exposure. Department 1 included high-risk exposure units such as the Emergency Department, Intensive Care Unit (ICU), Infectious Diseases, Respiratory and Critical Care Medicine, Fever Clinic, and Isolation Ward, where nurses face heavy workload intensity and direct contact with high-risk patients. Department 2 consisted of surgical and perioperative units, including General Surgery, Orthopedics, Urology, Thoracic Surgery, Neurosurgery, the Operating Room, and Burn & Plastic Surgery, characterized by fast-paced work and frequent night shifts. Department 3 encompassed internal medicine departments such as Cardiology, Nephrology, Gastroenterology, Hematology, Oncology, and Endocrinology, which typically involve prolonged management of chronic or critically ill patients. Department 4 included obstetrics, pediatrics, and other specialty units such as Obstetrics and Gynecology, the Delivery Room, Pediatrics, Neonatology, Rehabilitation, and Traditional Chinese Medicine, where high emotional demands are common. Department 5 comprised outpatient and medical-technical units, including outpatient clinics, Hemodialysis, Endoscopy, Interventional Radiology, Imaging, Laboratory, Pharmacy, and Intravenous Admixture Center, which usually operate with more regular work rhythms and comparatively lower psychological stress.

Sample size calculation

The required sample size was estimated using the simple random sampling formula with a 95% confidence level (Z = 1.96), an expected prevalence of 0.5, and a permissible error of 0.05 [10]. The minimum calculated sample size was 384, and considering a potential 10% invalid response rate, the final target sample size was set at no fewer than 420 participants.

Research instruments

General Information Questionnaire

The general information questionnaire was developed based on a comprehensive literature review and consultation with clinical and methodological experts. It captured key demographic and occupational variables, including sex, age, height, weight, marital status, educational level, annual income, working years, professional category, past medical history, and post-infection symptoms. These items were selected to ensure adequate characterization of participants and to allow exploration of potential associations with psychological outcomes.

Patient Health Questionnaire-9 (PHQ-9)

The PHQ-9 was used to assess the severity of depressive symptoms. The scale contains nine items, each scored from 0 to 3, with total scores ranging from 0 to 27. The PHQ-9 total score is classified into five levels: 0-4 (no depression), 5-9 (mild), 10-14 (moderate), 15-19 (moderately severe), and 20-27 (severe). Higher scores indicate more severe depression [11].

Generalized Anxiety Disorder-7 (GAD-7)

The GAD-7 scale was used to evaluate the severity of anxiety symptoms. It comprises seven items, each scored from 0 to 3, with total scores ranging from 0 to 21. The GAD-7 total score is classified into four levels: 0-4 (no anxiety), 5-9 (mild), 10-14 (moderate), and 15-21 (severe). Higher scores indicate greater anxiety severity [12].

Data collection

Data were collected via an online questionnaire distributed through the hospital’s internal network and email system. Participation was voluntary, and all responses were submitted anonymously. The questionnaire included the general information form and the PHQ-9 and GAD-7 scales. Logical consistency checks were embedded in the survey system to automatically identify and exclude invalid responses.

Statistical analysis

Data were double-checked, entered into Excel (Microsoft Corp., Redmond, WA, USA), and analyzed using IBM SPSS Statistics for Windows, Version 26 (Released 2018; IBM Corp., Armonk, New York, United States) and GraphPad Prism (Dotmatics, Boston, MA, USA). Continuous variables were presented as mean ± standard deviation (SD) or median (IQR), and categorical variables as frequencies and percentages. Group differences were analyzed using the t-test, Kruskal-Wallis test, or χ^2^ test, as appropriate. Variables with statistical significance in univariate analysis (P < 0.05) were included in multivariate logistic regression to identify independent factors associated with depression or anxiety, with results expressed as odds ratios (ORs) and 95% confidence intervals (CIs).

Quality control

Quality control was implemented throughout all stages of the study, including questionnaire design, distribution, data collection, and statistical analysis. The questionnaire was reviewed by domain experts and pre-tested before formal administration. Data entry was conducted by two independent researchers to ensure accuracy, and statistical analyses were independently performed by two analysts to verify the consistency and reliability of the results.

Ethical considerations

This study was conducted in accordance with the principles of the Declaration of Helsinki and was approved by the Ethics Committee of the participating hospital (Ethics Approval Number: 2024-SL-0046). All participants provided informed consent before enrollment and were free to withdraw from the study at any time without any impact on their work or rights. Data were used exclusively for research purposes and stored anonymously on a secure, access-controlled server. Participants who exhibited signs of psychological distress were offered counseling and referral services by the research team.

## Results

Baseline characteristics of participants

After excluding invalid and incomplete questionnaires, a total of 735 valid responses were included in the final analysis. The participants had a mean age of 30.12 ± 5.70 years, and the majority were female and married. Regarding departmental distribution, 95 (12.9%) worked in high-risk exposure departments, 175 (23.8%) in surgical and perioperative departments, 167 (22.7%) in internal medicine departments, 70 (9.5%) in obstetrics, pediatrics, and specialty departments, and 228 (31.0%) in outpatient and medical-technical departments. A total of 263 nurses reported persistent long COVID symptoms two years after infection (Table 1).

**Table 1:** Demographic and Clinical Characteristics of the Study Participants (n = 735) Values are presented as mean ± standard deviation (SD) or n (%).

Variable	Description	n (%)/Mean ± SD
Age (years)		30.1 ± 5.7
Sex	Male	36 (4.9)
	Female	699 (95.1)
Body mass index (BMI) (kg/m^2^)		22.21 ± 3.53
Education level	Associate degree	92 (12.5)
	Bachelor’s degree	633 (86.1)
	Master’s degree	10 (1.4)
Working years	0-5 years	245 (33.3)
	6-10 years	306 (41.6)
	11-20 years	150 (20.4)
	>21 years	34 (4.6)
Smoking	Yes	11 (1.5)
	No	724 (98.5)
Drinking	Yes	30 (4.1)
	No	705 (95.9)
Marriage	Single	265 (36.1)
	Married	465 (63.3)
	Divorced	5 (0.7)
Department (category)	Department 1	95 (12.9)
	Department 2	175 (23.8)
	Department 3	167 (22.7)
	Department 4	70 (9.5)
	Department 5	228 (31.0)
COVID status	Non-long COVID	472 (64.2)
	Long COVID	263 (35.8)
Generalized Anxiety Disorder-7 (GAD-7)		4.97 ± 4.80
Patient Health Questionnaire-9 (PHQ-9)		5.28 ± 4.90

Demographic and occupational differences between nurses with and without long COVID

Among the 735 participants, 263 nurses (35.8%) were identified as having long COVID, while 472 (64.2%) reported no persistent symptoms. Initial comparisons revealed notable differences between the two groups, prompting further analysis to explore the demographic and psychological characteristics associated with long COVID (Table 2). Nurses in the long COVID group were older and had significantly higher GAD-7 and PHQ-9 scores than those in the non-long COVID group, indicating higher levels of anxiety and depression. Regarding occupational characteristics, the distribution of working years differed significantly between groups: the long COVID group included fewer nurses with 0-5 years of experience but more with 6-10 years or 11-20 years of experience.

**Table 2:** Comparison of Demographic and Occupational Characteristics Between Nurses With and Without Long COVID (n = 735) Values are presented as mean ± standard deviation (SD), median (interquartile range (IQR)), or n (%). Statistical comparisons were conducted using the chi-square test, t-test, or Mann-Whitney U test, depending on data distribution. A p-value < 0.05 was considered statistically significant. GAD-7: Generalized Anxiety Disorder-7; PHQ-9: Patient Health Questionnaire-9

Variable	Description	Long COVID (n = 263)	Non-long COVID (n = 472)	P-value
Age (years)		31.08 ± 5.84	29.59 ± 5.56	0.0006
Sex	Male	18 (6.8)	18 (3.8)	0.068
	Female	245 (93.2)	454 (96.2)	
BMI (kg/m^2^)		22.50 ± 3.61	22.04 ± 3.49	0.0675
Education level	Associate degree	27 (10.3)	64 (13.8)	0.187
	Bachelor’s degree	234 (89.0)	399 (84.5)	
	Master’s degree	2 (0.8)	8 (1.7)	
Working years	0-5 years	74 (28.1)	171 (36.2)	0.005
	6-10 years	104 (39.5)	202 (42.8)	
	11-20 years	68 (25.9)	82 (17.4)	
	>21 years	17 (6.5)	17 (3.6)	
Smoking	Yes	6 (2.3)	5 (1.1)	0.322
	No	257 (97.7)	467 (98.9)	
Drinking	Yes	15 (5.7)	15 (3.2)	0.143
	No	248 (94.3)	457 (96.8)	
Marriage	Single	87 (33.1)	178 (37.7)	0.326
	Married	175 (66.5)	290 (61.4)	
	Divorced	1 (0.4)	4 (0.8)	
Department (category)	Department 1	33 (12.5)	62 (13.1)	0.378
	Department 2	59 (22.4)	116 (24.6)	
	Department 3	68 (25.9)	99 (21.0)	
	Department 4	29 (11.0)	41 (8.7)	
	Department 5	74 (28.1)	154 (32.6)	
GAD-7		6.71 ± 5.15	4.00 ± 4.30	<0.001
PHQ-9		7.34 ± 5.34	4.12 ± 4.23	<0.001

Multivariate logistic regression analysis of factors associated with long COVID

In the univariate analysis, several variables, including GAD-7, PHQ-9, age, sex, BMI, and working years, were found to be associated with long COVID. These factors were subsequently entered into a multivariate logistic regression model to determine which variables remained significant after adjustment. After stepwise selection, the PHQ-9 score and age remained in the final model (Table 3). The results showed that higher PHQ-9 scores (OR = 1.147, 95% CI = 1.108-1.188, P < 0.001) and older age (OR = 1.040, 95% CI = 1.011-1.070, P = 0.007) were independent risk factors for long COVID among nurses. The model demonstrated good overall fit (Nagelkerke R^2^ = 0.142; Hosmer-Lemeshow P > 0.05).

**Table 3:** Multivariate Logistic Regression Analysis of Factors Associated With Long COVID Values are presented as odds ratios (ORs) with 95% confidence intervals (CIs). Logistic regression was performed using variables with p < 0.10 in univariate analysis. A p-value < 0.05 was considered statistically significant. “-” indicates not applicable. PHQ-9: Patient Health Questionnaire-9; SE: standard error

Variable	β	SE	Wald	P-value	OR	95% CI for OR
PHQ-9	0.136	0.018	60.716	<0.001	1.147	1.108-1.188
Age	0.039	0.014	7.351	0.007	1.04	1.011-1.070
Constant	-2.518	0.45	31.359	<0.001	-	-

Comparison of demographic and psychological characteristics among departments

Across the five departmental categories, the incidence of long COVID did not differ significantly, indicating that the distribution of long COVID cases was relatively uniform across clinical settings. However, psychological indicators such as GAD-7 and PHQ-9 scores showed significant variation across departments, suggesting that work environments may influence emotional well-being even when infection risk is comparable (Table 4). Post hoc analysis using Tamhane’s T2 test showed that nurses in Department 1 had significantly higher GAD-7 and PHQ-9 scores than those in Department 5 (both P < 0.05), whereas no other pairwise departmental comparisons showed significant differences (P > 0.05). Overall, nurses in high-risk exposure departments tended to exhibit higher anxiety and depression levels compared with those in outpatient and technical departments.

**Table 4:** Comparison of Demographic, Psychological, and Outcome Variables Among Nurses by Department (n = 735) Values are presented as mean ± standard deviation (SD). Statistical analysis was performed using one-way analysis of variance (ANOVA), followed by Tamhane’s T2 post-hoc test due to unequal variances. A p-value < 0.05 was considered statistically significant. GAD-7: Generalized Anxiety Disorder-7; PHQ-9: Patient Health Questionnaire-9

Variable	Department 1	Department 2	Department 3	Department 4	Department 5	P-value
GAD-7	6.13 ± 5.44	4.94 ± 4.91	5.38 ± 4.81	4.63 ± 4.67	4.32 ± 4.36	0.024
PHQ-9	6.18 ± 4.69	5.48 ± 5.24	5.76 ± 5.18	5.19 ± 4.80	4.42 ± 4.44	0.016
Age (years)	29.73 ± 5.02	29.87 ± 5.60	30.75 ± 5.40	29.89 ± 5.66	30.07 ± 6.25	0.562
BMI (kg/m^2^)	22.52 ± 3.71	22.06 ± 3.14	22.27 ± 3.22	21.68 ± 3.42	22.30 ± 3.98	0.592
Working years	1.87 ± 0.82	1.97 ± 0.86	2.04 ± 0.84	1.94 ± 0.83	1.95 ± 0.88	0.671
Long COVID, n (%)	33 (34.7)	59 (33.7)	68 (40.7)	29 (41.4)	74 (32.5)	0.378

## Discussion

The present study found that nurses with long COVID were significantly older and exhibited higher GAD-7 and PHQ-9 scores compared with those without long COVID. Although statistically significant, these differences were clinically mild, as the mean scores in the long COVID group fell within the mild anxiety and mild depression ranges. These findings indicate that long COVID is associated with a substantial psychological burden among nursing staff, and they underscore the importance of identifying high-risk individuals within this population. Multivariate analysis further identified higher PHQ-9 scores and older age as factors independently associated with long COVID, suggesting that psychological distress and age-related physiological changes may be related to the presence and persistence of long COVID symptoms. Additionally, interdepartmental comparisons showed no significant difference in the incidence of long COVID across departments, implying a relatively even distribution among different clinical work settings. However, significant departmental differences were observed in GAD-7 and PHQ-9 scores, suggesting that variations in psychological stress among nurses may be influenced by job characteristics and working environments.

Comparison with previous studies

Previous studies have consistently indicated that healthcare workers are at high risk for long COVID, with more pronounced clinical and psychological sequelae than the general population [13]. Multiple systematic reviews have shown that within six months after infection, approximately one-third of healthcare professionals continue to report symptoms such as fatigue, insomnia, anxiety, or depression [14,15]. The findings of this study are generally consistent with those reports, further supporting the crucial role of psychological factors in the development and persistence of long COVID sequelae.

However, this study did not identify significant differences in the incidence of long COVID among nurses from different departments, which differs from some reports suggesting a higher risk among those working in intensive care, emergency, or infectious disease departments [16]. Possible explanations include (1) improved infection control systems and rotation mechanisms across departments in the later stages of the pandemic, leading to more balanced occupational exposure risks; (2) the development of similar psychological defense and coping mechanisms among nurses through prolonged pandemic response experience; and (3) relatively balanced sample sizes but limited within-group variability, which may have reduced the statistical power to detect intergroup differences.

Notably, this study revealed significant differences in anxiety and depression scores among nurses from different departments, with those in high-risk exposure departments showing markedly greater psychological burden compared with nurses in outpatient and medical-technical departments. This suggests that even when the infection risk is relatively uniform, the level of psychological stress may still vary depending on job characteristics and environmental intensity.

Nurses working in high-risk exposure departments, such as emergency, intensive care, infectious diseases, respiratory and critical care, fever clinics, and isolation wards, are often subjected to high-intensity shifts, emergency treatments, infection control pressure, and critically ill patients, all of which may trigger persistent anxiety and hypervigilance. In contrast, nurses in outpatient and medical-technical units, such as general outpatient services, dialysis centers, endoscopy and interventional suites, radiology, laboratories, and pharmacy or infusion centers, generally experience more controllable workloads and lower patient exposure risks, resulting in lighter psychological strain [17]. These findings suggest that nurses’ psychological responses are more strongly influenced by occupational exposure and job characteristics rather than by infection experience alone. Future interventions should therefore be tailored to specific departmental contexts, with targeted psychological support and stress management for high-exposure nurses to promote emotional recovery, professional resilience, and the sustainable well-being of the nursing workforce [18,19].

Possible mechanisms linking depression and age to long COVID

Depression and age were the two principal factors significantly associated with long COVID in this study. Their mechanisms of influence may involve dysregulation of immune-inflammatory responses, neuroendocrine imbalance, and chronic psychological stress.

Depression is not merely a psychological state but a systemic condition characterized by chronic immune activation and neuroinflammatory processes. Multiple studies have demonstrated that individuals with depression exhibit elevated proinflammatory cytokines, such as interleukin-6 (IL-6), tumor necrosis factor-α (TNF-α), and C-reactive protein (CRP), and decreased anti-inflammatory mediators. This inflammatory imbalance may delay post-viral tissue repair and sustain immune activation, thereby contributing to the persistence of long COVID symptoms [20,21]. Furthermore, depression affects the hypothalamic-pituitary-adrenal (HPA) axis and autonomic nervous system function, leading to elevated cortisol levels, circadian rhythm disruption, and metabolic dysregulation, all of which impair stress adaptation and resilience [22]. Behaviorally, depressive symptoms such as reduced physical activity, poor sleep quality, and diminished perceived social support may hinder recovery. The frequently observed pattern of “chronic fatigue-depression-immune activation” among long COVID patients supports this hypothesis [23,24].

With increasing age, the human immune system undergoes profound changes characterized by immunosenescence and inflammaging, including weakened adaptive immunity and heightened baseline inflammatory activity [25,26]. This immune state predisposes older adults to persistent inflammatory responses and delayed tissue repair following SARS-CoV-2 infection. In addition, endothelial dysfunction, mitochondrial energy impairment, and reduced neural plasticity have been implicated in the pathophysiology of sustained symptoms such as fatigue and cognitive dysfunction in long COVID [27-29].

Within the nursing workforce, older nurses frequently face heavier workloads, greater managerial responsibilities, chronic sleep deprivation, and circadian rhythm disruption-chronic stressors that may further impair immune regulation and diminish psychological recovery capacity. The interaction between physiological vulnerability and occupational stress exposure may therefore create a “dual susceptibility” of both mind and body, increasing the likelihood of persistent long COVID manifestations [30].

Importantly, an interactive amplification effect may exist between aging and depression. Age-related neuroinflammation and heightened HPA-axis reactivity increase biological susceptibility to depressive symptoms, while prolonged depression may, in turn, accelerate immune aging, metabolic dysfunction, and systemic inflammation - forming a vicious feedback loop of inflammatory, endocrine, and psychological dysregulation. This bidirectional interplay provides a plausible explanation for why older and more depressed nurses in this study were more likely to experience persistent long COVID symptoms [31,32].

Recommendations for clinical nurses in the context of the pandemic

The findings of this study show that although the incidence of long COVID did not differ significantly across departments, anxiety and depression levels varied markedly, and older age, together with higher depression scores, appeared to be factors independently associated with long COVID. These results highlight that in a prolonged pandemic context, equal attention should be given to both the physical and psychological recovery of nurses, alongside infection prevention and control.

A priority is to establish a risk-based psychological support system that incorporates mental health screening into routine occupational health management and records screening outcomes in health files. For high-risk individuals, such as those of older age, with elevated depression scores, or with a history of severe infection, priority follow-up should be arranged, including mental-health nursing interviews, anonymous counselling platforms, and accessible online self-assessment tools [33].

Improving work scheduling and rehabilitation mechanisms is also essential. Nurses who contract COVID-19 or develop long COVID may benefit from phased return-to-work plans that gradually reduce night shifts and high-intensity tasks. Regular monitoring of physical strength, sleep quality, and emotional well-being through a structured “rehabilitation assessment form,” as well as providing temporary rest or duty rotation for those with recurrent symptoms, can support recovery. Existing intervention studies have demonstrated that tailored psychological and wellness programmes effectively improve nurse well-being during the pandemic [34].

Strengthening professional resilience and fostering a supportive team culture further contribute to long-term psychological health. Continuing education on emotional regulation, peer-support activities, mentorship systems that pair experienced nurses with junior staff, and mindfulness-based practices may enhance team cohesion, reduce anxiety and burnout, and build sustained resilience [35,36].

Finally, developing a sustainable occupational health and rehabilitation framework is necessary to embed nurses’ physical and psychological well-being into routine quality and safety indicators. Leveraging hospital information systems to deliver periodic health questionnaires and constructing databases for dynamic monitoring and risk prediction can help shift nursing organizations from a focus solely on infection prevention toward holistic recovery and resilience.

Limitations

This study has several limitations that should be acknowledged. First, the use of a voluntary online survey introduces the possibility of selection and response bias. Because the response rate was not recorded, nurses experiencing persistent symptoms may have been more likely to participate, which could inflate both the estimated prevalence of long COVID and the strength of the observed associations. Second, the single-center, cross-sectional design limits the generalizability of the findings and prevents the determination of temporal sequence, raising the possibility of reverse causality. Third, long COVID was identified through self-reported symptoms without clinical confirmation, which may lead to misclassification or underestimation of mild or transient cases. In addition, psychological outcomes were assessed using self-reported instruments (GAD-7 and PHQ-9), which are subject to recall or reporting bias. Fourth, the sample consisted predominantly of female nurses, which may limit the applicability of the findings to more gender-balanced populations. Moreover, important confounding variables, such as social support, occupational stress, and sleep quality, were not included in the analysis and may have influenced psychological outcomes.

Despite these limitations, the study has several strengths. It focuses on nurses two years after COVID-19 infection, providing timely evidence on long-term psychological health in a high-risk occupational group. The use of validated mental health scales and a clearly defined long COVID classification enhances the robustness of the findings. Future studies should incorporate longitudinal follow-up, multi-center recruitment, documented response rates, and a broader range of psychosocial variables to improve generalizability and causal inference.

## Conclusions

This study revealed that nurses with long COVID were generally older and had significantly higher levels of anxiety and depression compared with those without long COVID. Multivariate analysis identified depressive severity (PHQ-9 score) and older age as independent risk factors. Although the prevalence of long COVID did not differ significantly across departments, variations in GAD-7 and PHQ-9 scores suggest that psychological burden is influenced by work-related factors. These findings highlight the need for continuous psychological monitoring and targeted interventions, particularly for older and high-risk nurses, to promote recovery and enhance occupational resilience in the post-pandemic era.
